# Is reciprocity really outcome-based? A second look at gift-exchange with random shocks

**DOI:** 10.1007/s40881-017-0041-2

**Published:** 2017-11-20

**Authors:** Brent J. Davis, Rudolf Kerschbamer, Regine Oexl

**Affiliations:** University of Innsbruck, Innsbruck, Austria

**Keywords:** Gift-exchange, Principal agent model, Incomplete contracts, Random shocks, Outcome-based reciprocity, Replication study, Laboratory experiment, C72, C91, D63, D81, H50

## Abstract

By means of a laboratory experiment, Rubin and Sheremeta (Manag Sci 62(4):985–999, 2016), study a bonus-version of the gift-exchange game, including two treatment variations. First they vary whether the effort provided by the agent directly translates into output for the principal, or whether it is distorted by a shock. Second, for the condition with a shock they vary whether the shock is observed by the principal, or not. The authors’ main findings are that (1) the introduction of an unobservable shock significantly reduces welfare; and (2) informing the principal about the size of the shock does not restore gift-exchange. In a replication study we largely reproduce finding (1), but we fail to confirm finding (2). Our data suggests that small behavioral differences in the initial rounds lead to a hysteresis effect that is responsible for the differences in results across studies.

**Electronic supplementary material:**

The online version of this article (10.1007/s40881-017-0041-2) contains supplementary material, which is available to authorized users.

## Introduction

Reciprocity has been shown to have the power to increase efficiency in labor-market relations governed by incomplete contracts—see Akerlof (1982) and Akerlof and Yellen (1988, 1990). In experimental economics, an important workhorse model to study reciprocity in labor-market relations is the gift-exchange game introduced by Fehr et al. (1993). The basic version of this game has two stages. In stage 1, a firm offers a contract—consisting of a wage and a desired effort—to a worker. In stage 2, the worker observes the contract chosen by the firm and makes an effort choice, knowing that effort increases the firm’s revenue but is personally costly to him. The game ends with the payoff of the firm increasing in the worker’s effort and decreasing in the wage, and the payoff of the worker increasing in the wage and decreasing in the effort.

Numerous studies have investigated variants of the gift-exchange game in lab experiments—see Fehr et al. (1993), Fehr et al. (1997), Fehr and Falk (1999), Charness (2000), and Gächter and Falk (2002), among others. Typically, workers provide more than the minimum effort, and effort is positively related to the wage (Fehr and Schmidt 2006; Charness and Kuhn 2011). As there is no direct material incentive for exerting more than the minimum effort in the one-shot version of the game, this finding is typically interpreted as evidence for reciprocity—workers reward the gift of a generous wage by giving a gift in the form of higher effort.

The impact of reciprocity has been shown to be even more pronounced in the bonus-version of the gift-exchange game. This version adds to the basic version an ‘adjustment’ stage, where the firm can reward or punish the worker for his performance at an own material cost, by increasing or decreasing the wage. Now both sides of the market have opportunities for reciprocal responses; this produces large and persistent increases in effort and thereby considerable efficiency gains—see Fehr et al. (1997, 2007).^1^

In the experiments by Fehr et al. (1997, 2007), the worker’s effort is perfectly observable by the firm. In a recent experiment, Rubin and Sheremeta (2016)—RS16 in the following—distort the worker’s effort choice in the bonus-version of the gift-exchange game by a random, zero-mean shock.

RS16 provide two competing hypotheses regarding the adjustment of the wage of the principal, depending on whether reciprocity is effort or outcome based. Effort-based reciprocity implies that the adjustment depends on the effort chosen by the worker, irrespective of the outcome; the opposite holds for outcome-based reciprocity where the outcome is the only thing that matters.^2^ RS16 find that (1) the introduction of an unobservable shock significantly reduces welfare; and (2) informing the principal about the size of the shock does not restore gift-exchange. These findings are inconsistent with effort-based reciprocity, but consistent with output-based reciprocity. Effort-based reciprocity implies that the zero-mean shock will reduce welfare only when effort is unobservable. The reason is that with effort-based reciprocity exerting more effort has the same consequences in an environment with an observable shock as in the setting without a shock. Moreover, under some plausible conditions, the effort in those two environments is higher than in a setting with an unobservable shock.^3^ In contrast, outcome-based reciprocity implies that the zero-mean shock will reduce welfare irrespective of whether it is observable or not. The reason is that with outcome-based reciprocity and convex costs the net marginal return of higher effort is lower in an environment with a shock as in a setting without a shock. Together these observations imply that effort-based and outcome-based reciprocity make qualitatively the same prediction regarding the comparison of the condition without a shock with the one with an unobservable shock (efficiency is lower in the latter than in the former), while they make different predictions regarding the comparisons involving the condition with an observable shock (effort-based reciprocity predicts that efficiency is the same as in the condition without a shock while outcome-based reciprocity predicts that it is the same as in the condition with an unobservable shock).

In our replication study, we implement the same three treatments as RS16: In the “No-Shock” treatment, the worker’s effort is not distorted by a shock. In the “Observable-Shock” treatment, the worker’s effort is distorted by a random, zero-mean shock; the principal observes both the effort and the shock before making her decision of how much to reward or punish the worker. The “Unobservable-Shock” treatment is like the Observable-Shock treatment, except that the principal only observes the outcome, which corresponds to the sum of effort plus shock.^4^

We largely reproduce RS16’s finding (1), but we fail to reproduce finding (2): Welfare is larger in the No-Shock treatment than in the Unobservable-Shock treatment, and it is statistically indistinguishable between the No-Shock treatment and the Observable-Shock treatment (while in RS16 welfare is larger in the No-Shock treatment than in the Observable-Shock treatment).

While the pattern of results presented by RS16 is in line with outcome-based reciprocity and our evidence is in line with effort-based reciprocity (see the explanations above) this fact alone is not really indicative of different types of reciprocity being at work in the two studies. To receive more information we analyzed the decisions in the adjustment stage in the two studies more closely. Based on our data we find that the principal’s adjustment in the Observable-Shock treatment is increasing in the shock (in line with the hypothesis that reciprocity is at least partly outcome-based), but also that the impact of the agent’s effort on the adjustment is more pronounced and more robust than that of the shock (consistent with the hypothesis that reciprocity is mainly effort-based). Also, an increase in effort has the same effect on the adjustment in the No-Shock and the Observable-Shock treatment—in line with effort-based reciprocity. We therefore conclude that the evidence from the adjustment stage in our data is consistent with the hypothesis that with observable effort, reciprocity is mainly effort-based. Looking at the adjustment stage in the RS16 data we find very similar patterns of wage adjustments as in our data in all the treatments. There is one notable exception: In round 1 of the Observable-Shock treatment of RS16 there are exceptionally many observations consistent with outcome-based reciprocity but inconsistent with effort-based reciprocity—by fare more than in any other round of the same treatment in the same study and by far more than in round 1 of our Observable-Shock treatment. We will argue later that this difference in first round behavior in the Observable-Shock treatment might have produced a hysteresis effect that is responsible for the differences in results across studies.

## Experimental design

Our design replicates that of RS16. The game consists of 10 periods, each period having three stages. In stage one, the principal (she) offers a contract $(w,e^*)$, consisting of a wage $w\in \{1,2,\ldots ,100\}$ and an (unenforceable) desired effort $e^*\in \{0,1,\ldots ,14\}$ that she would like the agent to undertake. In stage two, the agent (he) observes the contract chosen by the principal and decides about the effort level $e\in \{0,1,\ldots ,14\},$ knowing that the cost of effort, *c*(*e*),  is $e^{2}/2$, rounded to the next highest integer. In the No-Shock treatment the outcome *y* is simply the effort *e*. In the treatments with a shock (Observable-Shock and Unobservable-Shock) the outcome is the effort plus an integer component $\epsilon$ (i.e., $y=e+\epsilon$), which is uniformly distributed on $\{-2,-1,0,1,2\}$.^5^ In stage three, the principal observes either only the effort (No-Shock), or only the outcome (Unobservable-Shock), or both (Observable-Shock), and chooses an adjustment *a* from the set $\{-50,-40,\ldots ,0,\ldots ,40,50\}$. Payoffs are $\pi ^{P}=10y-w-\frac{|a|}{10}$ for the principal (adjustment is costly to the principal) and $\pi ^{A}=w-c(e)+a$ for the agent. Details are common knowledge among all participants; i.e. they know the payoff structure, the set of actions available to each player at each stage, and in the treatments with shock they know the size and the probabilities of all possible shock levels.

The experiment was programmed in z-Tree (Fischbacher 2007); participants were recruited via hroot (Bock et al. 2014). Roles were fixed and participants were randomly re-matched each period. Sessions were run in 2016 at the Innsbruck EconLab, lasting around 70 min. On average, participants earned €12.96.

We ran three sessions per treatment; with three matching groups of eight per session (four principals, four agents), creating nine independent observations per treatment.^6^ Given the difference in the mean effort between the No-Shock and the Observable-Shock treatment—the two treatments where effort is observable—in RS16’s data, and given the respective standard deviations, with a sample size of nine observations in each condition and an $\alpha$ of 5%, we have a power of 88% (*t* test).^7^

## Results

We are mainly interested in welfare—defined as the sum of the payoffs of the two parties. Since effort and adjustment are the variables determining welfare, we start by presenting the main result in terms of welfare, and then present the evidence regarding the two components of welfare in backward induction order (i.e., starting with stage 3). In the online-appendix, we extend the analysis by also including wage and desired effort and by displaying the corresponding values of RS16 (see Table 4 in the online-appendix).

**Table 1:**
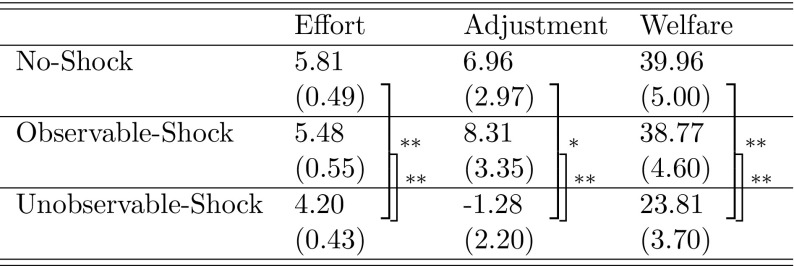
Summary statistics

Standard errors in parentheses are based on 9 independent observations; stars for significance according to Mann–Whitney *U* tests, based on 9 independent observations: $$^{*}$$ $$p<0.10$$, $$^{**}$$ $$p<0.05$$, $$^{***}$$ $$p<0.01$$

**Fig. 1:**
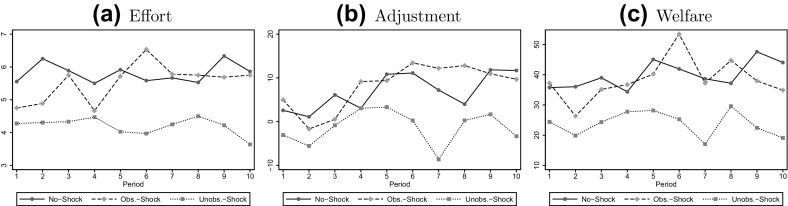
Averages per period

### Welfare

Average welfare is summarized in Table 1. It is higher in the No-Shock than in the Unobservable-Shock treatment (Mann–Whitney *U* (MWU) test, based on 9 independent observations in each condition: *p* = 0.05) and it is higher in the Observable-Shock than in the Unobservable-Shock treatment (MWU test: *p* = 0.04). However, it does not differ between the No-Shock and the Observable-Shock treatment (MWU test: *p* = 0.96). Panel (c) of Fig. 1 and a panel regression controlling for period effects (see Table 5 in the online-appendix) confirm these results.^8^

**Result 1** *In line with findings of RS16, the introduction of an unobservable shock significantly reduces welfare compared to the treatment without shock. In contrast to the findings of RS16, welfare is significantly larger when the shock is observable rather than unobservable, and statistically indistinguishable between the treatment with observable shock and the one without shock.*

### Adjustment

**Table 2:** Adjustment across the no-shock and observable-shock treatment

Treatment	Av. adjustment and # of obs.			MWU tests across deviations, *p*-values		
	$e < e^*$	$e = e^*$	$e > e^*$	$e < e^*$ vs. $e > e^*$	$e < e^*$ vs. $e = e^*$	$e > e^*$ vs. $e = e^*$
No-Shock	− 5.42	19.97	22.26	< 0.01	< 0.01	0.66
*N*	189	121	50			
Observable-Shock	− 4.09	24.48	23.57	< 0.01	< 0.01	0.69
*N*	193	87	56			
MWU tests across treatments, *p*-values						
	0.90	0.15	0.54			

*e*: effort; $e^*$: desired effort

Table 1 shows the average adjustment for each treatment; Table 2 shows the average adjustment for given differences between effort (*e*) and desired effort ($e^*$) for the two treatments where effort is observable. In both treatments, effort choices that negatively deviate from the desired effort are punished. By contrast, positive deviations of effort from desired effort are hardly rewarded more than exact fulfillment. This is in line with findings in the literature, indicating that with observable effort, reciprocity is effort-based and that negative reciprocity is a more powerful and robust behavioral phenomena than positive reciprocity—see Abbink et al. (2000), Fehr and Gächter (2000), Baumeister et al. (2001), and Charness and Kuhn (2011).

We find no significant difference between the No-Shock and the Observable-Shock treatment in adjustment for effort being greater, equal, or lower to desired effort (MWU tests: all *p*-values $\ge$ 0.15). This suggests that matching the desired effort and deviating from it is rewarded or punished similarly in the two treatments. Panel regressions controlling for period effects and demographic variables confirm these results—see Table 6 in the online-appendix.

**Table 3:** Observable-shock treatment: adjustment for a given shock level

	Av. adjustment and # of obs.			MWU tests across deviations, *p*-values		
Shock	$e < e^*$	$e = e^*$	$e > e^*$	$e < e^*$ vs. $e > e^*$	$e < e^*$ vs. $e = e^*$	$e > e^*$ vs. $e = e^*$
$\epsilon < 0$	− 7.00	21.95	16.36	< 0.01	< 0.01	0.33
*N*	80	41	22			
$\epsilon = 0$	− 8.37	26.43	24.55	< 0.01	< 0.01	0.98
*N*	43	14	11			
$\epsilon > 0$	1.86	26.88	30.00	< 0.01	< 0.01	0.85
*N*	70	32	23			
MWU tests across shocks, *p*-values						
$\epsilon < 0$ vs. $\epsilon > 0$	0.02	0.48	0.06			
$\epsilon < 0$ vs. $\epsilon = 0$	0.98	0.62	0.22			
$\epsilon = 0$k vs. $\epsilon > 0$	0.07	0.99	0.81			

*e*: effort; $e^*$: desired effort; $\epsilon$: shock

Table 3 dis-aggregates the average adjustment in the Observable-Shock treatment in the effects of negative, zero, or positive deviations of effort from desired effort for different shock levels ($\epsilon$); it provides support for the hypothesis that the adjustment is influenced by the size of the shock. The effect is most pronounced for positive and negative deviations of effort from desired effort, but (even there) the overall impact seems moderate: When effort exceeds the desired effort, the average adjustment is 16.36 after a negative shock but 30.00 after a positive shock (MWU test: *p* = 0.06). When effort is below the desired effort, the adjustment is −7.00 after a negative shock, but 1.86 after a positive shock (MWU test: *p* = 0.02). Importantly, although these two differences are significant at conventional levels when considered in isolation, none of the significant results in the bottom part of Table 3 survives a Bonferroni correction for the simultaneous testing of 18 (or even only 9) hypotheses.^9^

We also searched in other ways for evidence in support of outcome-based reciprocity. For instance, given that we mainly find evidence for negative reciprocity, outcome-based reciprocity would imply that punishments that are unjustified when reciprocity is effort-based (i.e., negative adjustments for effort $\ge$ desired effort) occur more frequently in the Observable-Shock than in the No-Shock treatment – simply because negative shocks happen in the former but are impossible in the latter treatment. This is not what we find, though: When effort is (weakly) larger than desired effort, the frequency of punishments is 4% in the No-Shock but only 2% in the Observable-Shock treatment (two-sample test of proportions: *p* = 0.32). By contrast, in environments where unjustified punishments are unavoidable even under effort-based reciprocity (because effort is not observable in the Unobservable-Shock treatment), they happen more frequently. The frequency of negative adjustments for positive deviations of effort from desired effort in the Unobservable-Shock treatment is 8%, and the difference in frequencies between the Unobservable-Shock and the Observable-Shock treatment is significant (two-sample test of proportions: *p* = 0.02).

Turning to the impact of the agent’s effort on the adjustment, we find economically more pronounced and statistically more robust results: The differences in adjustments between negative deviations of effort from desired effort and zero deviations exceed 20 units for all shock levels and all significant differences displayed in the right part of Table 3 remain significant even when correcting them for the simultaneous testing of 18 hypotheses.

Overall, the evidence presented in Table 3 seems to be consistent with effort-based negative reciprocity, plus some forgiving when the effect of the negative deviation of effort from desired effort is cushioned by a positive shock: When effort is lower than desired effort, the principal punishes the agent with a negative adjustment, except for the case when the negative deviation comes together with a positive random shock.^10^ We summarize our findings in the following result:

**Result 2** *While the adjustment in the Observable-Shock treatment is influenced by the size of the shock, the impact of effort on the adjustment is more pronounced and more robust than that of the shock. Also, the impact of effort on the adjustment is similar in the Observable-Shock and the No-Shock treatment.*

### Effort

Average effort is summarized in Table 1. It is higher in the No-Shock than in the Unobservable-Shock treatment (MWU test: *p* = 0.02), and it is higher in the Observable-Shock than in the Unobservable-Shock treatment (MWU test: *p* = 0.03). It does not differ between the No-Shock and the Observable-Shock treatment (MWU test: *p* = 0.83). Panel (a) of Fig. 1 and a panel regression including period effects (see Table 8 in the online-appendix) confirm these results.^11^ For the relationship between wage and effort, see Table 9 in the online-appendix.

**Result 3** *In line with our findings for welfare, average effort is higher in the No-Shock than in the Unobservable-Shock treatment, and higher in the Observable-Shock than in the Unobservable-Shock treatment. It is statistically indistinguishable between the treatment with observable shock and the one without shock, however.*

## Discussion

We have reproduced RS16’s finding that the introduction of shocks reduces efforts when shocks are unobserved. However, we failed to confirm the result that observable shocks have the same impact on behavior as unobservable shocks. Indeed, our evidence from the adjustment stage of the game is consistent with the hypothesis that with observable shocks, reciprocity is mainly effort-based, and that on the top of effort-based reciprocal responses principals share part of the windfall profits (or losses) generated by the shock with the agent. Participants seem to anticipate that the behavior in the last stage of the game is qualitatively similar in the Observable-Shock and the No-Shock treatment. They therefore behave similarly in these two treatments also earlier in the game.

Our finding that an observable random shock does not impair gift-exchange is consistent with recent evidence from laboratory experiments analyzing employer-employee relationships in the face of exogenous shocks (Kocher and Strasser 2011; Gerhards and Heinz 2017). It is also in line with recent results from “noisy” public goods games with sanctioning mechanisms.^12^ However, it is not in line with the main result of RS16. What drives the differences in results?

One candidate explanation is subject pool effects: While RS16 have conducted their study in the US, our sessions have taken place in Austria. If factors such as culture and experience affect gift-exchange—as suggested by the results in Charness et al. (2004), for instance—then reciprocity might be more outcome-based in the subject pool investigated by RS16 than in ours; this tendency could potentially explain the differences in results. We searched for evidence that points in that direction, but failed to find such evidence. Indeed, when we reconstruct our Table 3 using RS16’s data, we find results that are very similar to those reported in Sect. 3.2; reconstructing our Table 7 with RS16’s data we also find similar results—see Table 10 in the online-appendix.

A second candidate explanation is differences in learning dynamics across studies. Comparing the time trends in the Observable-Shock treatment across studies, we find that the difference in average effort is small in the first round but increases steadily in later rounds—for the sake of comparison, we have included RS16’s figures in the online-appendix (Fig. 3). The following differences in dynamics are responsible for the increasing gap: In our study, the effort in the Observable-Shock treatment is initially between the corresponding values in the No-Shock and the Unobservable-Shock treatment. However, after some ‘learning rounds’, average effort in the Observable-Shock treatment converges to the increasing path in the No-Shock treatment, while in the Unobservable-Shock treatment it stays rather constant at a lower level—see panel (a) of Fig. 1. By contrast, in RS16, the effort in all the treatments is initially roughly the same. After the initial round, the average effort in the No-Shock treatment increases over rounds, while it stays rather constant at a lower level in the Observable-Shock and the Unobservable-Shock treatment—see panel (a) of Fig. 3 in the online-appendix.

The differences in the time trends of effort provision in the Observable-Shock treatment are confirmed by panel regressions controlling for wage, desired effort and ‘inverse period’—see Table 9 in the online-appendix. While the effects of wage and desired effort on effort are comparable across studies for all the treatments—and the effect of ‘inverse period’ on effort is comparable across studies for the No-Shock and the Unobservable-Shock treatment—there is a different time trend in the Observable-Shock treatment: while effort is significantly increasing over periods in our study, the variable ‘inverse period’ is insignificant in RS16’s data.

What causes these differences in dynamics across studies? To address this question we investigated the behavior of participants in the initial round. While negative adjustments for cases where effort weakly exceeds desired effort are *generally* rather rare (2% in Observable-Shock, 4% in No-Shock, and 8% in Unobservable-Shock in our data), they occur quite frequently in *round one* of the Observable-Shock treatment in RS16. Specifically, in round one of the Observable-Shock treatment the frequency of such ‘unjustified punishments’ is 33% in RS16’s data, but only 7% in our data (two sample test of proportions: *p* = 0.08). This high frequency of unjustified punishments in round one in RS16 might have led agents to believe that exerting high effort is not an adequate shelter against punishment. Such beliefs might have reduced their effort in subsequent rounds. With lower effort in subsequent rounds agents forgo the opportunity to learn that principal behavior is very similar in the Observable-Shock and the No-Shock treatment.

We conclude that in settings characterized by strategic complementarities, small differences in the behavior in initial rounds (caused by subject pool effects or by pure chance) might lead to differences in learning dynamics and, thereby, to path-dependent outcomes in the long run. This insight is not specific to the context under consideration or to lab experiments more generally—it is rather a well-known phenomenon (often called ‘hysteresis’) in many branches in economics and beyond. Turning back to the specific context of gift-exchange with random shocks, we conclude that although our results are plausible, their robustness has to be verified in future experiments before policy conclusions can be drawn from existing evidence.

## Electronic supplementary material

Below is the link to the electronic supplementary material.

Supplementary material 1 (pdf 971 KB)

## Footnotes

1 Throughout the paper we follow large parts of the experimental economics literature in using the terms ‘efficiency’ and ‘welfare’ interchangeably for the sum of the material payoffs of the two parties involved.

2 In the treatment with an observable shock, effort-based reciprocity is in line with intention-based reciprocity—as modeled, among others, by Rabin (1993) and Dufwenberg and Kirchsteiger (2004)—while outcome-based reciprocity is not. This is so because (1) with intention-based reciprocity second-order beliefs are used to assess the intentions of others, which are then rewarded or punished; and (2) with outcome-based reciprocity a negative shock is punished even if the agent exerts high effort although higher effort increases the payoff of the principal independently of her second-order belief.

3 For the environment with an unobservable shock effort-based reciprocity boils down to outcome-based reciprocity because output is the only signal for effort in this case. Since the signal is noisy and costs are convex the net marginal return of higher effort is lower in this environment than in environments where effort is observable.

4 Our No-Shock treatment corresponds to “Effort-Only” in RS16, the Observable-Shock treatment corresponds to “Effort-Shock”, and the Unobservable-Shock treatment corresponds to “Outcome-Only”.

5 Note that the range of the shock does not depend on the level of effort exerted by the worker. As a consequence, if the worker chooses a very low level of effort in a given round and if the shock in the respective round is negative, the output might be negative, too. To address the issue of negative payoffs, subjects were informed at the very beginning that losses will be subtracted from the show up fee (of €9).

6 In one session of the Observable-Shock treatment, we had a virus appearing at one computer in period five. For the affected group, observations for periods five to ten and demographic information was not stored.

7 We assume an asymptotically normal distribution for this power calculation—even though with just nine observations per condition this might not strictly hold and we might have somewhat less power. Note also that our sample size is the same as in RS16.

8 Following RS16, we estimate panel models with individual participants representing random effects and standard errors clustered at the matching group level and calculated via bootstrap. We have also estimated these panel models controlling for gender, age, and risk aversion; results remain unaffected.

9 In panel regressions controlling for period effects and demographic variables the shock has a significant impact, yet it is lower than the impact of an additional unit of effort (see Table 7 in the online-appendix).

10 Reference-dependent preferences (as modeled by Köszegi and Rabin 2006, for instance) can help account for this behavior when the output resulting from the desired effort level constitutes some kind of reference point for the principal. In the treatment with deterministic output, effort below the desired effort is punished because it implies an output below the reference point. In the Observable-Shock treatment effort below the desired effort is not punished if it is accompanied by a positive shock because the reference point in terms of outcome is still reached in this case.

11 Panel (a) of Fig. 1 suggests that in the first four periods average effort is lower in the Observable-Shock than in the No-Shock treatment. The difference between the two treatments is not significant, however. This is true for each single period (MWU-tests: all *p*-values > 0.16) and for the average of the first four periods (MWU test: *p* = 0.25).

12
Grechenig et al. (2010), for instance, investigate the impact of sanctions in a setting where there is uncertainty about the contributions of others. Their findings suggest that sufficient information accuracy about others’ behavior is crucial for efficiency. Xiao and Kunreuther (2016) investigate a social dilemma where actions are observable but outcomes are a stochastic function of actions. They find that punishment is still effective although non-cooperative behavior is less likely to be punished in such an environment. In an earlier contribution, Ambrus and Greiner (2012) find in a repeated public goods game that in a perfect monitoring environment, increasing the severity of the potential punishment monotonically increases efficiency, while with imperfect monitoring the relationship is U-shaped.
