# Supplementary material for: Is reciprocity really outcome-based? A second look at gift-exchange with random shocks
Source: J Econ Sci Assoc. 2017 Nov 20;3(2):149–60. doi: 10.1007/s40881-017-0041-2 (PMC6956943; doi:10.1007/s40881-017-0041-2)
Supplement: Supplementary file 1 — Supplementary material 1 (pdf 971 KB) [file 40881_2017_41_MOESM1_ESM.pdf]

A Online-appendix

Table 4: Averages of all decision variables and payoffs, our and RS16's data

	Wage	Desired effort	Effort	Adjustment	Principal's payoff	Agent's payoff	Welfare
**Our data**							
No-Shock	35.19	8.08	5.81	6.96	20.68	19.28	39.96
	(3.07)	(0.52)	(0.49)	(2.97)	(2.47)	(3.49)	(5.00)
Observable-Shock	37.31	7.66	5.48	8.31	14.92	23.85	38.77
	(3.29)	(0.34)	(0.55)	(3.35)	(2.94)	(3.43)	(4.60)
Unobservable-Shock	29.21	6.44	4.20	−1.28	10.10	13.71	23.81
	(3.00)	(0.44)	(0.43)	(2.20)	(3.18)	(2.66)	(3.70)
**RS16's data**							
No-Shock	41.14	8.95	6.40	1.44	20.91	17.01	37.92
	(3.22)	(0.31)	(0.43)	(3.41)	(3.11)	(2.77)	(4.28)
Observable-Shock	33.45	7.63	4.69	−1.81	11.23	14.78	26.01
	(2.98)	(0.34)	(0.34)	(2.21)	(3.46)	(3.28)	(3.41)
Unobservable-Shock	33.85	7.63	4.69	−4.97	12.04	13.16	25.19
	(2.28)	(0.25)	(0.41)	(1.32)	(2.85)	(1.95)	(2.33)

Standard errors in parenthesis are based on 9 indep. observations; stars for significance according to Mann-Whitney U-tests, based on 9 indep. observations: * $p < 0.10$, ** $p < 0.05$, *** $p < 0.01$

Figure 2: Average per period, first stage behavior

(a) Wage (b) Desired effort

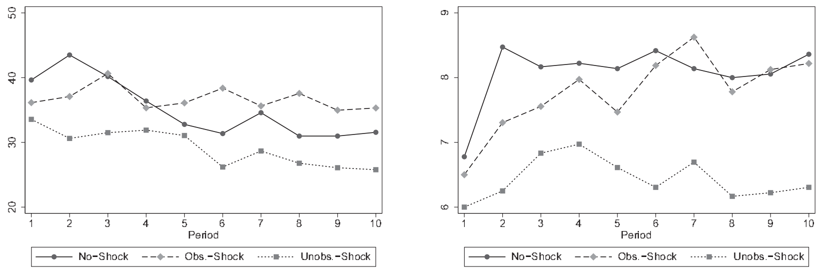

Figure 3: Average per period – RS16's data

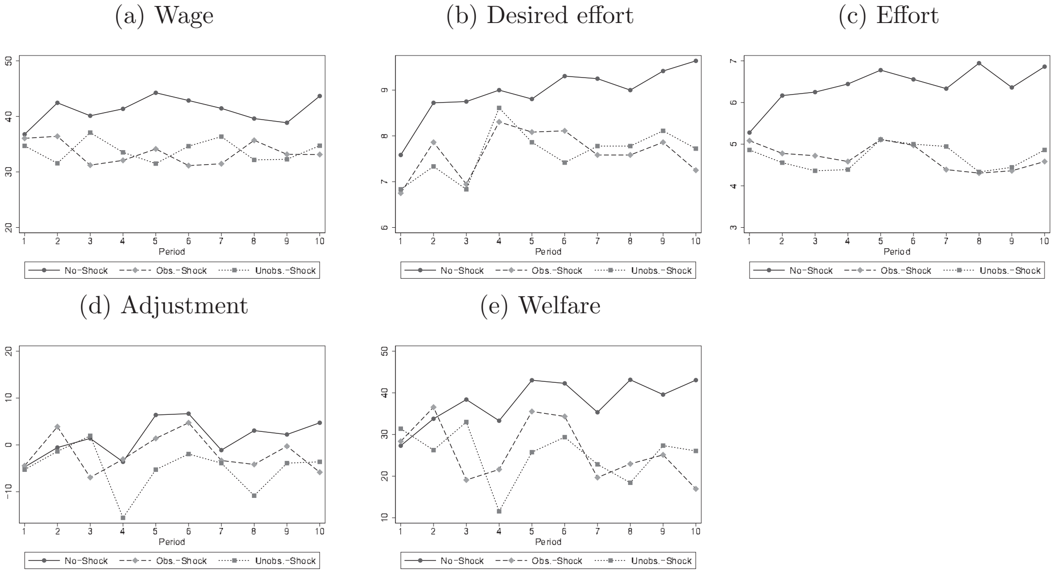

Table 5: Panel model of welfare, controlling for differences between treatments

	(1)	
	All treatments	
	Dep. var.: welfare	
Observable-Shock	−1.37	(8.40)
Unobservable-Shock	-18.65^{***}	(7.20)
Inverse period	−8.40	(7.92)
Observable-Shock x inv. period	−0.11	(12.08)
Unobservable-Shock x inv. period	8.51	(10.57)
Constant	42.42^{***}	(5.57)
Observations	1056	

Standard errors in parentheses are clustered on the group level and calculated via bootstrap; * $p < 0.10$, ** $p < 0.05$, *** $p < 0.01$. Observable-Shock = 1 if treatment = 'Observable-Shock', zero otherwise. Unobservable-Shock = 1 if treatment = 'Unobservable-Shock' and zero otherwise. Inv. period runs from 1 to 1/10. χ^2-test on difference between the treatment dummies: $p = 0.03$.

Table 6: Panel models of adjustment, controlling for differences between treatments

	(1) No-Shock & Observable-Shock		(2) No-Shock & Observable-Shock		(3) No-Shock & Observable-Shock		(4) No-Shock & Observable-Shock	
	Dependent variable: adjustment							
No-Shock (NS)	−0.60	(4.24)	−2.13	(3.75)	0.79	(4.31)	−0.70	(3.87)
Effort – des. effort	3.28***	(0.88)	1.70**	(0.76)	3.34***	(0.89)	1.84**	(0.80)
NS x Effort – des. effort	0.17	(1.05)	−0.08	(0.96)	0.40	(1.03)	0.14	(0.93)
Effort > des. effort			0.15	(3.69)			1.00	(3.66)
Effort < des. effort			−19.07***	(3.14)			−17.81***	(3.24)
Inverse period					−13.99***	(4.72)	−13.32***	(4.71)
Gender					4.27	(3.34)	3.50	(3.23)
Age					0.54	(0.36)	0.49	(0.38)
Constant	15.37***	(3.01)	22.75***	(3.15)	4.62	(9.27)	12.55	(10.73)
Observations	696		696		670		670	

Standard errors in parentheses are clustered on the group level and calculated via bootstrap; * $p < 0.10$, ** $p < 0.05$, *** $p < 0.01$. 'Effort > des. effort' is a dummy = 1 if effort > des. effort, zero otherwise; 'Effort < des. effort' is a dummy = 1 if effort < des. effort, zero otherwise. No-Shock (NS) is a dummy if treatment = 'No-Shock', zero otherwise. Inv. period runs from 1 to 1/10.

Table 7: Panel models of adjustment, investigating importance of shock and effort

	(1) Observable-Shock		(2) Observable-Shock		(3) Observable-Shock		(4) Observable-Shock	
	Dependent variable: adjustment							
Effort – des. effort	3.27***	(0.87)	1.67**	(0.81)	3.35***	(0.84)	1.92**	(0.90)
Shock	2.65***	(0.72)	2.56***	(0.68)	3.34***	(0.55)	3.24***	(0.56)
Effort > des. effort			−1.82	(6.74)			2.36	(6.11)
Effort < des. effort			−19.78***	(4.67)			−15.87***	(3.90)
Inverse period					−16.43***	(5.98)	−14.69***	(5.17)
Gender					5.98	(4.05)	5.45	(3.86)
Age					0.97	(0.60)	0.96	(0.73)
Constant	15.53***	(2.96)	23.59***	(4.52)	−5.49	(15.37)	−0.09	(18.30)
Observations	336		336		320		320	

Standard errors in parentheses are clustered on the group level and calculated via bootstrap; * $p < 0.10$, ** $p < 0.05$, *** $p < 0.01$. 'Effort > des. effort' is a dummy = 1 if effort > des. effort, zero otherwise; 'Effort < des. effort' is a dummy = 1 if effort < des. effort, zero otherwise. Inv. period runs from 1 to 1/10.

Table 8: Panel model of effort, controlling for differences between treatments

	(1) All treatments	
	Dep. var.: effort	
Observable-Shock	0.05	(0.89)
Unobservable-Shock	−1.71**	(0.76)
Inverse period	−0.13	(0.55)
Observable-Shock x inv. period	−1.18	(0.82)
Unobservable-Shock x inv. period	0.36	(0.70)
Constant	5.85***	(0.55)
Observations	1056	

Standard errors in parentheses are clustered on the group level and calculated via bootstrap; * $p < 0.10$, ** $p < 0.05$, *** $p < 0.01$. Observable-Shock = 1 if treatment = 'Observable-Shock', zero otherwise. Unobservable-Shock = 1 if treatment = 'Unobservable-Shock' and zero otherwise. Inv. period runs from 1 to 1/10. χ^2-test on difference between the treatment dummies: $p = 0.05$.

Table 9: Panel model of effort, our data and RS16 data

	(1)	(2)	(3)	(4)	(5)	(6)
		Our Data			RS16 Data	
	No-Shock	Observable-Shock	Unobservable-Shock	No-Shock	Observable-Shock	Unobservable-Shock
	Dependent variable: effort					
Wage	0.07***	0.07***	0.07***	0.09***	0.07***	0.06***
	(0.01)	(0.01)	(0.01)	(0.00)	(0.01)	(0.01)
Desired effort	0.11**	0.05	0.05	0.10**	0.07	0.09
	(0.05)	(0.05)	(0.03)	(0.04)	(0.06)	(0.06)
Inverse period	−0.80**	−1.20**	−0.32	−0.93**	0.39	0.15
	(0.34)	(0.52)	(0.43)	(0.36)	(0.65)	(0.47)
Constant	2.69***	2.88***	1.77***	2.06***	1.59***	1.85***
	(0.38)	(0.66)	(0.39)	(0.34)	(0.35)	(0.41)
Observations	360	336	360	360	360	360

Standard errors in parentheses are clustered on the group level and calculated via bootstrap; * $p < 0.10$, ** $p < 0.05$, *** $p < 0.01$. Inv. period runs from 1 to 1/10.

Table 10: Panel models of adjustment, investigating importance of shock and effort – RS16's data

	(1)		(2)		(3)		(4)	
	Observable-Shock		Observable-Shock		Observable-Shock		Observable-Shock	
	Dependent variable: adjustment							
Effort – des. effort	2.22***	(0.53)	1.47***	(0.50)	2.24***	(0.55)	1.52***	(0.48)
Shock	2.24***	(0.80)	2.33***	(0.78)	2.35***	(0.80)	2.42***	(0.79)
Effort > des. effort			−1.56	(4.88)			−1.28	(4.50)
Effort < des. effort			−10.97***	(3.32)			−10.37***	(3.24)
Inverse period					−5.40	(3.83)	−4.11	(3.62)
Gender					−2.95	(4.57)	−3.03	(4.38)
Age					1.49	(1.30)	1.18	(1.31)
Constant	4.87*	(2.91)	10.54***	(3.88)	−17.46	(27.39)	−6.41	(26.89)
Observations	360		360		360		360	

Standard errors in parentheses are clustered on the group level and calculated via bootstrap; * $p < 0.10$, ** $p < 0.05$, *** $p < 0.01$. 'Effort > des. effort' is a dummy = 1 if effort > des. effort, zero otherwise; 'Effort < des. effort' is a dummy = 1 if effort < des. effort, zero otherwise. Inv. period runs from 1 to 1/10.

B Instructions

[[[These are the instructions for the Observable-Shock treatment. When instructions are adapted to the No-Shock or the Unobservable-Shock treatment, we mark the respective parts with squared brackets.]]]

Dear participants,

welcome to today's experiment. For today's experiment, funds are provided by the Austrian Science fund.

Please read the instructions for the experiment carefully. All statements in the instructions are true, and all participants receive exactly the same instructions. Your earnings in the experiment depend on your decisions and potentially the decisions of others. If you have a question, please raise your hand. Your question will then be answered privately. The experiment as well as the data analysis is anonymous. For a better understanding, in the following we will only use male designations. Those should be understood gender neutral.

We ask you to not talk to other participants and to use only the resources and devices that are provided by the conductors of the experiment. Please switch off all electronic devices. In addition, at the computer you are only allowed to use features that are necessary for the experiment. If you do not comply with these rules, you won't be paid in this experiment and you are not allowed to participate in any further experiments.

The currency used in the experiment is tokens. Tokens will be converted to Euros at a rate of 10 tokens to 1 Euro. You have already received a €11.00 participation fee. Your earnings from the experiment will be incorporated into your participation fee. At the end of today's experiment, you will be paid in private and in cash.

The experiment consists of two parts. Together, both parts will last for around 75 minutes. The two parts of the experiment are completely independent from each other. That is, your payment for part x only depends on decisions that you take in part x, and does not depend on decisions you take in the other part of the experiment. At the beginning of each part you receive the corresponding instructions. We will read the instructions out loud and will give you time for questions.

Thank you a lot for your attention and for participating in today's experiment.

Part 1

YOUR ROLE ASSIGNMENT

This part consists of 10 periods. Each period, you will be randomly and anonymously placed into a group which consists of two participants: participant A and participant B. At the beginning of the first period you will be randomly assigned either as participant A or participant B. You will remain in the same role throughout part 1 of the experiment. So, if you are assigned as participant B then you will stay as participant B throughout the entire part 1. Each consecutive period you will be randomly re-grouped with another participant of opposite assignment. So, if you are participant B, each period you will be randomly re-grouped with another participant A.

Each period will proceed in three stages.

STAGE 1

In stage 1, participant A will choose a reward (any integer number between 0 and 100) and a desired effort (any integer number between 0 and 14) for participant B.

An example of the stage 1 decision screen for participant A is shown below.

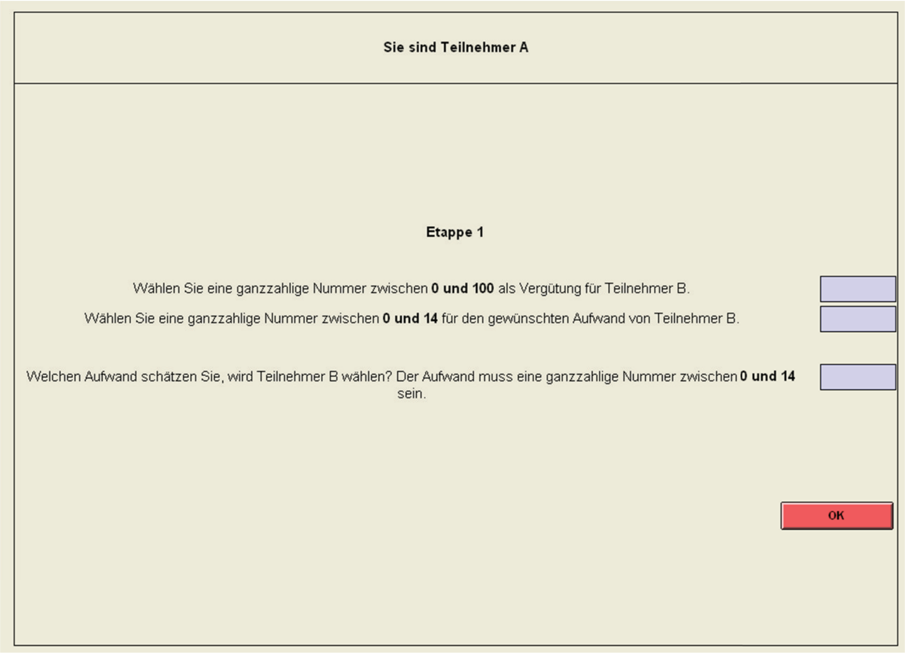

STAGE 2

On the screen, participant B is shown the reward and the desired effort chosen by participant A. Then, participant B will choose an effort level (any integer number between 0 and 14).

An example of the stage 2 decision screen for participant B is shown below.

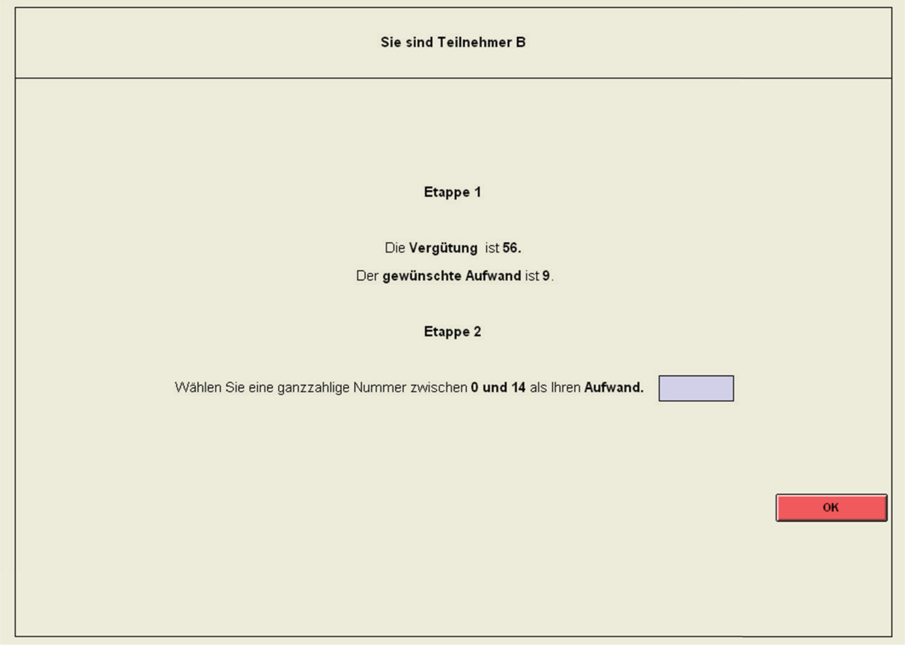

For each effort level chosen by participant B there is an associated cost of effort. The cost of effort can be found in the following table: Note that as effort rises from 0 to 14, costs rise

Effort	0	1	2	3	4	5	6	7	8	9	10	11	12	13	14
Cost of effort	0	1	2	5	8	13	18	25	32	41	50	61	72	85	98

exponentially.

STAGE 3

After participant B chooses the effort level, the computer will add to effort a random number to determine the performance of participant B:

Participant B's performance = effort + random number.

The random number chosen by the computer can take a value of -2, -1, 0, 1, or 2. Each number is equally likely to be drawn. After the computer makes the draw of a random number, it will display to participant A the performance of participant B on the screen, as well as the effort chosen by participant B and the random number chosen by the computer.

[[[No-Shock treatment: After participant B chooses the effort level, the performance of participant B is determined as follows: Participant B's performance = effort of participant B. Then the computer will display to participant A the performance of participant B on the screen.]]]

[[[Unobservable-Shock treatment: After participant B chooses the effort level, the computer will add to effort a random number to determine the performance of participant B: Participant B's performance = effort + random number. The random number chosen by the computer can take a value of -2, -1, 0, 1, or 2. Each number is equally likely to be drawn. Following the draw of the random number Participants B's performance will be shown to Participant A. Participant A will not know Participant B's actual effort or the random number drawn by the computer. Then, in the third stage, participants 1 will choose an adjustment level. The adjustment level must be a multiple of 10, between -50 and 50.]]]

An example of the stage 3 decision screen for participant A is shown below.

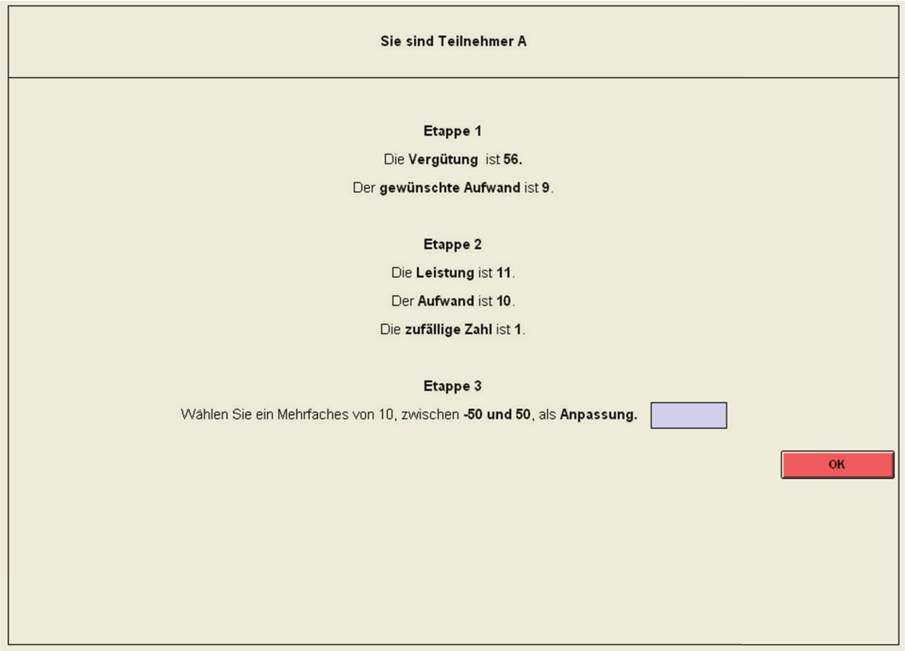

For each adjustment level chosen by participant A there is an associated cost of adjustment. The cost of adjustment can be found in the following table:

Adjustment	-50	-40	-30	-20	-10	0	10	20	30	40	50
Cost of adjustment	5	4	3	2	1	0	1	2	3	4	5

EARNINGS OF PARTICIPANT A

The earnings of participant A depend on the reward chosen by participant A in the first stage, the performance of participant B in the second stage and the adjustment chosen by participant A in the third stage. Specifically, the participant A's earnings are calculated by the following formula:

Participant A's earnings = 10*(performance participant B) – (reward) – (cost of adjustment)
= 10*(effort of participant B + random number) – (reward) – (cost of adj.)

[[[No-Shock
Participant A's earnings = 10*(performance participant B) – (reward) – (cost of adjustment)]]]

[[[Unobservable-Shock
Participant A's earnings = 10*(performance participant B) – (reward) – (cost of adjustment)
= 10*(effort of participant B + random number) – (reward) – (cost of adj.)]]]

Note that higher participant B's effort implies higher participant B's performance, and thus higher participant A's earnings. On the other hand, a higher reward or a higher cost of adjustment implies lower participant A's earnings.

EARNINGS OF PARTICIPANT B

The earnings of participant B depend on the reward chosen by participant A in the first stage, the cost of the effort chosen by participant B in the second stage and the adjustment chosen by participant A in the third stage. Specifically, participant B's earnings are calculated by the following formula:

Participant B's earnings = (reward) – (cost of effort) + (adjustment)

Note that a higher reward chosen by participant A implies higher participant B's earnings. On the other hand, a higher effort implies higher effort costs and therefore lower participant B's earnings. If participant A chooses a positive adjustment level for participant B then participant B's earnings increase by that adjustment level. However, if participant A chooses a negative adjustment level then participant B's earnings decrease by that adjustment level.

Example 1

Assume the following scenario. In the first stage, participant A chooses a reward of 50 and a desired effort of 7. In the second stage, participant B chooses an effort of 6. Then the computer randomly selects 2 as a random number, so the performance of participant B is 8 (6+2). Then the computer displays to participant A that participant B's performance is 8, participant B's effort is 6 and the random number chosen by the computer is 2. After observing this information, in the third stage, participant A chooses an adjustment of -40.

Therefore, participant A's earnings = 10*8 – 50 – 4 = 26, since participant B's performance is 8, the reward is 50, and the cost of adjustment of -40 is 4. Finally, participant B's earnings = 50 – 18 – 40 = -8, since the reward is 50, the cost of effort of 6 is 18, and the adjustment is -40.

Example 2

Assume the following scenario. In the first stage, participant A chooses a reward of 40 and a desired effort of 6. In the second stage, participant B chooses an effort of 9. Then the computer randomly selects -2 as a random number, so the performance of participant B is 7 (9-2). Then the computer displays to participant A that participant B's performance is 7, participant B's effort is 9 and the random number chosen by the computer is -2. After observing this information, in the third stage, participant A chooses an adjustment of 30. Therefore, participant A's earnings = 10*7 – 40 – 3 = 27, since participant B's performance is 7, the reward is 40, and the cost of adjustment of 30 is 3. Finally, participant B's earnings = 40 – 41 + 30 = 29, since the reward is 40, the cost of effort of 9 is 41, and the adjustment is 30.

[[[The examples are accordingly adjusted for the No-Shock and Unobservable-Shock treatments.]]]

END OF THE PERIOD

The computer will display to both participants the following information: the reward chosen by participant A, the desired effort chosen by participant A, the performance of participant B, the effort chosen by participant B, the random number chosen by the computer, the adjustment chosen by participant A, as well as individual earnings for that period.

[[[No-Shock: At the end of each period, the computer will display to both participants the following information: the reward chosen by participant A, the desired effort chosen by participant A, the performance of participant B, the adjustment chosen by participant A, as well as individual earnings for that period. An example of the outcome screen is shown below.]]]

[[[Unobservable-Shock: The computer will display to both participants the following information: the reward chosen by participant A, the desired effort chosen by participant A, the performance of participant B, the effort chosen by participant B, the random number chosen by the computer, the adjustment chosen by participant A, as well as individual earnings for that period.]]]

At the end of each period, the computer will calculate individual earnings.

An example is shown on the following picture.

Once your earnings are displayed on the outcome screen as shown below you should record your earnings for the period on your Personal Record Sheet under the appropriate heading.

IMPORTANT NOTES

Remember you have already received a €11.00 participation fee. In part 1 of the experiment, depending

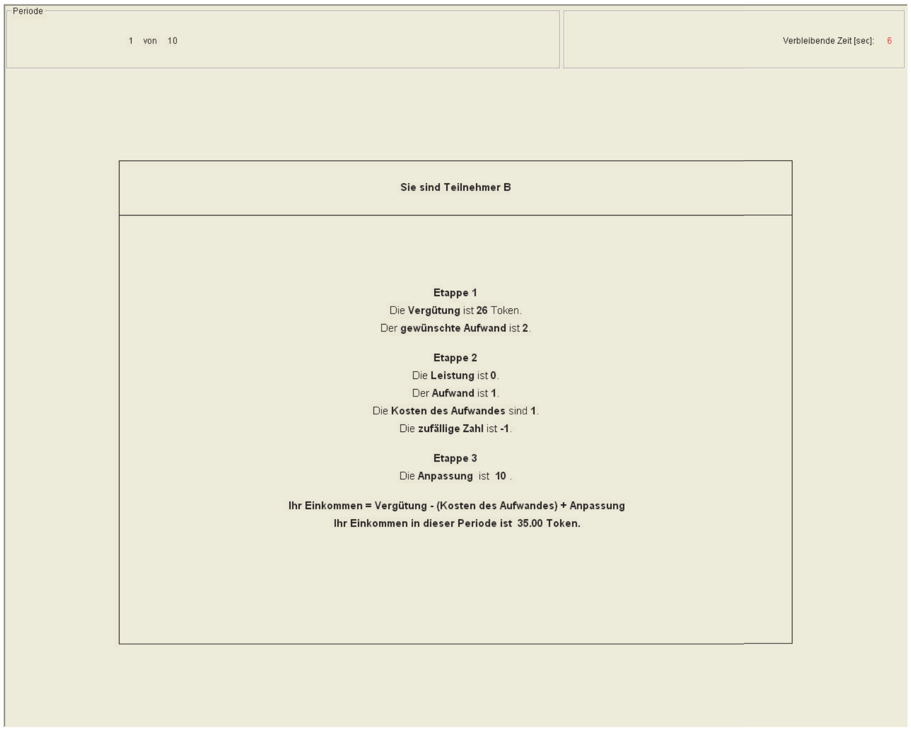

on a period, you may receive either positive or negative earnings. At the end of part 1 we will randomly select 1 out of 10 periods for actual payment and convert the income thereof to a payment in Euros. If the earnings are negative, we will subtract them from your total earnings. If the earnings are positive, we will add them to your total earnings. Are there any questions?

Control questions (implemented in z-Tree)

Question 1: Assume the following scenario. In the first stage, participant A chooses a reward of 30 and a desired effort of 8. In the second stage, participant B chooses an effort of 7. Then the computer selects 1 as a random number, so the performance of participant B is 8 (7 +1). Then the computer displays to participant A that participant B's performance is 8, participant B's effort is 7 and the random number chosen by the computer is 1. After observing this information, in the third stage, participant A chooses an adjustment of 40. What are participant A's earnings? _____(correct: 10*8-30-4 = 46) What are participant B's earnings? _____ (correct: 30-25+40 = 45)

Question 2: Assume the following scenario. In the first stage, participant A chooses a reward of 40 and a desired effort of 5. In the second stage, participant B chooses an effort of 1. Then the computer selects -1 as a random number, so the performance of participant B is 0 (1 -1). Then the computer displays to participant A that participant B's performance is 0, participant B's effort is 1 and the random number chosen by the computer is -1. After observing this information, in the third stage, participant A chooses an adjustment of -50. What are participant A's earnings? _____(correct: 10*0-40 -5 = -45) What are participant B's earnings? _____ (correct: 40-1-50 = -11)]]]

[[[No-Shock, Question 1: Assume the following scenario. In the first stage, participant A chooses a reward of 30 and a desired effort of 8. In the second stage, participant B chooses an effort of 7, so the performance of participant B is 7. Then the computer displays to participant A that participant B's performance is 7. After observing this information, in the third stage, participant A chooses an adjustment of 40. What are participant A's earnings? _____(correct: 10*7-30-4 = 36) What are participant B's earnings? _____ (correct: 30-25+40 = 45)

Question 2: Assume the following scenario. In the first stage, participant A chooses a reward of 40 and a desired effort of 5. In the second stage, participant B chooses an effort of 1, so the performance of participant B is 1. Then the computer displays to participant A that participant B's performance is 1. After observing this information, in the third stage, participant A chooses an adjustment of -50. What are participant A's earnings? _____(correct: 10*1-40-5 = -35) What are participant B's earnings? _____ (correct: 40-1-50 = -11)]]]

[[[Unobservable-Shock, Question 1: Assume the following scenario. In the first stage, participant A chooses a reward of 30 and a desired effort of 8. In the second stage, participant B chooses an effort of 7. Then the computer selects 1 as a random number, so the performance of participant B is 8 (7 + 1). Then the computer displays to participant A that participant B's performance is 8. After observing this information, in the third stage, participant A chooses an adjustment of 40. What are participant A's earnings? _____(correct: 10*8-30-4 = 46) What are participant B's earnings? _____ (correct: 30-25+40 = 45)

Question 2: Assume the following scenario. In the first stage, participant A chooses a reward of 40 and a desired effort of 5. In the second stage, participant B chooses an effort of 1. Then the computer selects -1 as a random number, so the performance of participant B is 0 (1 – 1). Then the computer displays to participant A that participant B's performance is 0. After observing this information, in the third stage, participant A chooses an adjustment of -50. What are participant A's earnings? _____(correct: 10*0-40-5 = -45) What are participant B's earnings? _____ (correct: 40-1-50 = -11)]]]

Personal Record Sheet

This sheet is for yourself, to record the earnings you have for each period.

You are participant A / B (circle one)

Period	Step 1 Wage	Step 1 Desired effort	Step 2 Perform.	Step 2 Effort	Step 2 Random number	Step 3 Adj.	Income from decisions/beliefs	Income period
1								
2								
3								

. . .

Total income:
Payment of part 1:
Income of the chosen period in token:
Income of the chosen period in Euro: ______ (1)
Participation fee: ______ (2)
Payment of part 2: ______ (3)
Total payment: (1) + (2) + (3) ____

Part 2

On your computer screen you will see a square composed of 100 numbered boxes, like shown below.

1	2	3	4	5	6	7	8	9	10
11	12	13	14	15	16	17	18	19	20
21	22	23	24	25	26	27	28	29	30
31	32	33	34	35	36	37	38	39	40
41	42	43	44	45	46	47	48	49	50
51	52	53	54	55	56	57	58	59	60
61	62	63	64	65	66	67	68	69	70
71	72	73	74	75	76	77	78	79	80
81	82	83	84	85	86	87	88	89	90
91	92	93	94	95	96	97	98	99	100

Behind one of these boxes hides a mine; all the other 99 boxes are free from mines. You do not know where this mine lies. You only know that the mine can be in any place with equal probability.

Your task is to decide how many boxes to collect. Boxes will be collected in numerical order. So you will be asked to choose a number between 1 and 100.

At the end of the experiment we will randomly determine the number of the box containing the mine. If you happen to have harvested the box where the mine is located – i.e. if your chosen number is greater than or equal to the drawn number – you will earn zero. If the mine is located in a box that you did not harvest – i.e. if your chosen number is smaller than the drawn number – you will earn in euro an amount equivalent to the number you have chosen.

In the next screen we will ask you to indicate how many boxes you would like to collect. You confirm your choice by hitting ‘OK’.

Extra Sheet

Effort participant B	0	1	2	3	4	5	6	7	8	9	10	11	12	13	14
Cost of effort	0	1	2	5	8	13	18	25	32	41	50	61	72	85	98

Adjustment participant A	-50	-40	-30	-20	-10	0	10	20	30	40	50
Cost of adjustment	5	4	3	2	1	0	1	2	3	4	5

Participant B's performance = effort + random number.

Participant A's earnings = 10*(performance participant B) – (reward) – (cost of adjustment)

= 10*(effort of participant B + random number) – (reward) – (cost of adj.)

[[[No-Shock

Participant A's earnings = 10*(performance participant B) – (reward) – (cost of adjustment)]]]

[[[Unobservable-Shock

Participant A's earnings = 10*(performance participant B) – (reward) – (cost of adjustment)

= 10*(effort of participant B + random number) – (reward) – (cost of adj.)]]]

Participant B's earnings = (reward) – (cost of effort) + (adjustment)
